# Gymnasiums

**Published:** 1860-05

**Authors:** 


### GYMNASIUMS.

WHAT is the use of eating like a pig, and then have to work like a "slave" to get rid of it, or explode? The best gymnasium is a wood-yard, a "clearing," or a corn-field. There is some sense in these things, because a valuable object is accomplished by the efforts, and the healthful influence of the same thrown in, thus killing two birds with one stone, which is Nature's method of procedure in many beautiful instances. The saliva, the tear-drop, and the perspiration, lubricate the mouth, and eye, and skin, and at the same time carry out from the body a large proportion of its waste and impurity. The breath which comes from the lungs is so loaded down with the *débris* of the system, that if inhaled in the state in which it leaves the body, it would produce instantaneous death; so impure, that if kept a single minute longer in the lungs than ordinary, we fairly gasp for life; and yet, that same foul breath, under the name of carbonic acid gas, makes, in its outward passage, the soft whisper from beauty's lips, the ravishing notes of delicious music, or the thunder tones of resistless oratory.

Suppose a fellow learns in time, and by labor enough to earn a small farm, to climb a greased pole fifty feet high, what is he to do when he gets there but to slide back in double quick time to the place he started from, and then go about his business?

What if he can jump sky-high, or turn a dozen somersets without stopping, or lift a calf bigger than himself, or hold, at arms' length, for two or ten minutes, a heavier weight than his own soggy head, what does he get by the "operation"? We hear of some "doctor" going about the country lifting up enormous weights, and exhibiting feats of strength which make a practical man feel what a pity he wasn't employed in felling trees, or mauling rails, or grubbing potatoes. It is stated that he has lifted with his hands a weight of one thousand one hundred and thirty-six pounds, and that he was sanguine, in twenty days more, of being able to lift twelve hundred pounds. The more he can prove himself to lift, the bigger fool he is, and the more fit for an asylum; for the next thing will be that he has ruptured a blood-vessel, and then for the remainder of life he won't be able to earn his salt, and some body will have to support him.

It is reported that arrangements are in progress for establishing gymnasiums for students, and the members of Young Men's Associations. Are our embryo doctors, and lawyers, and clergymen, going to make Tom Hyers and Bill Pooles and Yankee Sullivans of themselves? Does the ability of a jurist depend on the amount of beef he carries? Is a physician's skill to be determined by the hardness of his muscles? Is a clergyman's efficiency measured by the agility of his monkey capers, by his dexterity in hanging on to a beam by his hind-leg, and swinging up to touch his nose against the big toe of "'tother foot"?

A man's intellectuality does not depend on the amount of brute force which he possesses. It does not require a giant's strength to write a sermon, or make a book, or "clear" a thief, or feel a pulse. Of an assembly of French *savans*, on a certain occasion, Humboldt, being present, was found, by an accurate mode of measurement, to have the least muscular strength of the whole company, of which he was the greatest and the oldest. Small men, fragile men, men of little muscular vigor may have good bodily health, and among such are found a vast excess in numbers of the opposite class, and in all ages and countries who are the brightest of the world's bright stars. As a very general rule, it holds good—the bigger the man the bigger fool is he. Whoever saw a giant who was remarkable for any thing beyond the size of his body; while the smallness of his head, and the little that is in it, is a notable thing. Both body and brain need vital force; the mind is great in proportion as that vital force is expended in the brain, but if it is used up in developing the muscles, the brain must suffer. If one expects to make his living by the exercise of muscular strength, let him, as a boy and a youth, develop that strength by steady labor, and a regular and temperate life; if it is his wish to make money by legerdemain, by monkey capers, by rope-walking, by miraculous poises, and astonishing feats of ground and lofty tumbling, then the gymnasium is a very proper place for him, and it is well that the energies of the system should be expended in the direction of the muscles; but if he aims at a professional life, one which is to be followed as a means of living, he must exercise the mental, not the muscular, powers; to the brain, and not to the beef, must the energies of the system be sent, in order that, by their exercise, the brain may be developed, and the mind work with power.

To sedentary persons, violent, sudden, and fitful exercise is always injurious, and such are gymnastic performances. Soldiers die early. To-day they are doing nothing—to-morrow the forced march, the terrible battle summon up to the very dregs the employment of dormant energies. The disabilities and death of a campaign are many time greater by disease than by the bullet, for shocks, great alternations, always cause disease.

The exercise of the student should be regular, gentle, deliberate, always stopping short of felt fatigue. One hour's joyous walk with a cheerful friend in street, or field, or woodland, will never fail to do a greater and a more unmixed good, than double the time in the most scientifically conducted gymnasium in the world. There are individual cases where the gymnasium is of the most undeniable benefit, but the masses would be the better for having nothing to do with them. A million times better recipe than the gymnasium for sedentary persons, is:

Eat moderately and regularly of plain nourishing food well prepared.

Spend two or three hours every day in the open air regardless of the weather, in moderate, untiring activities.

